# The switch from conventional to atypical antipsychotic treatment should not be based exclusively on the presence of cognitive deficits. A pilot study in individuals with schizophrenia

**DOI:** 10.1186/1471-244X-10-47

**Published:** 2010-06-15

**Authors:** Gabriel Selva-Vera, Vicent Balanzá-Martínez, José Salazar-Fraile, José Sánchez-Moreno, Anabel Martinez-Aran, Patricia Correa, Eduard Vieta, Rafael Tabarés-Seisdedos

**Affiliations:** 1 the Teaching Unit of Psychiatry and Psychological Medicine, Department of Medicine, University of Valencia, Blasco-Ibáñez 17, 46010 Valencia, Spain; 2 Ciber en Salud Mental (CIBERSAM). Instituto de Salud Carlos III, Madrid, Spain; 3 the Bipolar Disorders Program, Clinical Institute of Neuroscience, Hospital Clinic of Barcelona, IDIBAPS, University of Barcelona, Villarroel 170, 08036 Barcelona, Spain

## Abstract

**Background:**

Atypical antipsychotics provide better control of the negative and affective symptoms of schizophrenia when compared with conventional neuroleptics; nevertheless, their heightened ability to improve cognitive dysfunction remains a matter of debate. This study aimed to examine the changes in cognition associated with long-term antipsychotic treatment and to evaluate the effect of the type of antipsychotic (conventional *versus *novel antipsychotic drugs) on cognitive performance over time.

**Methods:**

In this naturalistic study, we used a comprehensive neuropsychological battery of tests to assess a sample of schizophrenia patients taking either conventional (*n *= 13) or novel antipsychotics (*n *= 26) at baseline and at two years after.

**Results:**

Continuous antipsychotic treatment regardless of class was associated with improvement on verbal fluency, executive functions, and visual and verbal memory. Patients taking atypical antipsychotics did not show greater cognitive enhancement over two years than patients taking conventional antipsychotics.

**Conclusions:**

Although long-term antipsychotic treatment slightly improved cognitive function, the switch from conventional to atypical antipsychotic treatment should not be based exclusively on the presence of these cognitive deficits.

## Background

Cognitive disturbances are a core feature of schizophrenia and have been extensively studied in recent years [1]. Cognitive impairment is present before the onset of the illness [2] and is also found in healthy relatives of patients, although to a lesser degree [3]. In addition, this feature is not exclusively secondary to psychiatric symptoms or medication [4]. Cognitive impairment is a better predictor of future functional outcomes compared with positive symptoms [5-7].

The positive action of conventional antipsychotics drugs (APDs) on cognition is considered mild or moderate [8] and is limited to certain cognitive domains such as sustained attention [9,10].

Regarding novel antipsychotics, this supposed cognitive enhancement would be mediated by their capability to raise the level of dopamine and acetylcholine in prefrontal regions [11]. However, their different affinity for brain receptors may result in different procognitive profiles of each class of antipsychotics. Many studies support a cognitive enhancement of the different atypical antipsychotics: quetiapine and olanzapine [12], quetiapine and risperidone [13], ziprasidone and olanzapine [14]; olanzapine, quetiapine, and risperidone [15], risperidone and quetiapine focusing in schizophrenia with predominantly negative symptoms [16]. Moreover, this favorable effect on cognition seems to persist after controlling for confounding variables such as clinical state, learning, and cooperation [17]. Nevertheless, some authors [18] have reported a worsening in a working memory task in first episode schizophrenic patients after treatment with atypical antipsychotics.

Studies that attempted to demonstrate the association of a greater cognitive enhancement with novel versus conventional APDs are susceptible to biases and face several difficulties, which include confounding effects of clinical symptoms, previous and adjunctive medications (i.e., anticholinergics and benzodiazepines), and practice effects, especially when intervals between assessments are short [19,20].

Many studies have compared the procognitive properties of conventional and atypical APDs. Results that stemmed from these various reports were compiled by Woodward et al. [21] in a meta-analysis of 14 typical/atypical comparative studies; these authors concluded that atypical APDs improve overall cognitive function to a higher degree than typical APDs.

Despite all these data, the benefits of atypical APDs over conventional APDs for the full range of cognitive disturbances in schizophrenia remain controversial, especially for the most severe impairments, i.e., serial learning, executive functioning, vigilance, motor speed, and verbal fluency [22]. Several authors suggest that the difference in potency between these two APD types, if any, is small [19]. Recently, results of the neurocognitive component of the Clinical Antipsychotic Trials of Intervention Effectiveness (CATIE), which is a NIMH-sponsored study, did not indicate a better procognitive profile of four atypical APDs when compared with the conventional APD perphenazine [23].

Most of the studies comparing typical versus atypical antipsychotics on cognitive performance are randomized blind trials. Since many clinicians consider changing from conventional to novel antipsychotics in order to enhance cognition, we think that naturalistic studies may provide a closer look to real clinical practice.

In the present naturalistic, retrospective study, we used a comprehensive neuropsychological battery of tests to assess the cognitive outcome in two groups of schizophrenic patients: the first group was treated with one or more conventional APDs over two years, while the second group was treated with one atypical APD. The treatments were not discontinued at any time and there were no switches in the type of APD administered. Patients' performance on tasks of executive functions, verbal working memory, short-term memory, verbal memory, visual memory, speed of processing, verbal fluency, and motor speed were assessed twice, two years apart.

The first objective of this study was to examine changes in cognitive impairment associated with long-term APD treatment. The second objective was to assess the effect of the type of antipsychotic treatment on change of cognition over two years and describe the potential differences between the groups.

## Methods

### Subjects

Subjects who participated in this observational and naturalistic study were enrolled in the Valencia Follow-Up Study of Schizophrenia and Bipolar I Disorder [24-26]. Fifty-two patients who fulfilled the DSM-IV criteria for schizophrenia [27] were recruited over nine months among individuals attending three psychiatric outpatient units in Valencia, Spain. Diagnoses were confirmed by the Schedules for Clinical Assessment in Neuropsychiatry (SCAN) and the CATEGO computer program [28] after a minimum disease progress of two years. The cohort of patients was divided into two groups according to the type of antipsychotic treatment. Only patients who took the same type of medication (conventional or atypical APDs) and were not hospitalized for the entire two years were evaluated. Changes in dosage were permitted. Thirty-nine patients from the original cohort of schizophrenic individuals met these criteria. One group (*n *= 13, 9 men, 4 women) was composed of patients being treated with conventional antipsychotics (fluphenazine, *n *= 7; haloperidol, *n *= 2; perphenazine, *n *= 1; and haloperidol plus fluphenazine, *n *= 3) and the second group (*n *= 26, 18 men, 8 women) comprised patients who were taking an atypical antipsychotic (olanzapine, *n *= 13; risperidone, *n *= 7; and quetiapine, *n *= 6).

All participants were assessed at baseline (T1) and two years later (T2). Most patients in this study were treated in settings that did not offer psychosocial rehabilitation programs. At each time point, all patients were medicated by their psychiatrists in a naturalistic manner. The average daily dose of APD was converted into chlorpromazine equivalent (CPZ) units, for statistical purposes [29,30]. The doses of benzodiazepines were converted to diazepam equivalent units. Biperiden was the only anticholinergic drug administered to the patients.

Written informed consent was obtained from all participants after an explanation of the study procedures. The Ethics Committee of the University Clinic Hospital of Valencia approved the research protocol.

### Clinical assessment

The clinical evaluation of each patient was rated according to the Positive and Negative Symptom Scale (PANSS) [31,32] and to the Hamilton Rating Scale for Depression (HRSD) [33,34]. Premorbid adjustment was assessed using the Phillips Adjustment Scale [35].

### Neuropsychological evaluation

All patients completed a battery of tests, which were described in three previous publications [36,7,37]. These tests were used to measure eight neurocognitive domains, in the following sequence. This sequence was always the same for all patients and took 90 minutes approximately in one experimental session.

1) Executive Functions/Reasoning and Problem Solving (Wisconsin Card Sorting Test [WCST] measuring Categories, Total errors and Perseverative Errors; Trail Making Test part B; and Color-Word Interference Trial of the Stroop Color and Word Test).

2) Short-term Memory (Digit Span Forward Test)

3) Working Memory (backward part of the Digit Span Test from the Wechsler Adult Intelligence Scale-Revised [WAIS-R]).

4) Verbal Memory (Babcock Story Recall Test)

5) Visual Memory (Rey-Osterrieth Complex Figure Test). Immediate and differed (30 minutes) recall.

6) Visual-Motor Processing/Speed of Processing (Trail Making Test part A; Digit Symbol Substitution Test [DSST] from the WAIS-R).

7) Semantic Verbal Fluency (FAS Test from the Controlled Oral Word Association Test and the Category Instant Generation Test [CIG]).

8) Motor Speed (Finger-Tapping Test in unimanual and bimanual conditions).

The variable years of education was used as a measure of premorbid intelligence.

### Data analyses

Data analyses were carried out using the SPSS software (version 15.0 for Windows). An alpha level of 0.05 was used for all statistical tests. Data were analyzed using Student's paired *t *test to compare the means (T1 vs. T2) for all patients, as well as for each treatment group at T1. In each group, changes in cognitive and clinical scores were analyzed using an analysis of variance (ANOVA) with repeated measures. Cognitive performance at T1 and T2 were the dependent variables and the type of APD was the independent variable. As CPZ units and PANSS positive scores at T1 were the only variables differing between that reached statistical significance, they were entered as covariates in these analyses.

Pearson correlations were calculated among the clinical, treatment, and outcome variables at T1 and for the difference in neurocognitive scores (T2-T1) for every cognitive variable. This new variable was calculated to better assess the evolution of cognitive performance. Two sets of correlation analyses were carried out by splitting the cohort of patients according to the type of antipsychotic medication taken.

Linear regression analyses with a forward stepwise procedure were performed to assess the relative contributions of the variables cited above. In this model, the type of antipsychotic medication and the clinical, outcome, and treatment variables at baseline that significantly correlated with neurocognitive measures (*P *≤ 0.05) in any of the two correlations were entered in the regression models as independent variables. The dependent variable was the T2-T1 difference in performance on each neuropsychological test.

## Results

### General characteristics of patients

The total sample comprised 27 men and 12 women. The mean age of all patients was 32.9 (SD [standard deviation] = 8.30) years, the mean length of education was 10.1 (SD = 3.01) years, the mean age at onset was 24.94 (SD = 7.05) years, and the mean number of prior episodes was 2.25 (SD = 1.59). The mean dose of APDs was 773.97 (SD = 514.63) CPZ units and the mean dose of anticholinergic medication (biperiden) was 0.53 mg (SD = 1.33). The mean dose of benzodiazepines was 3.55 (SD = 8.94) diazepam equivalent units. At baseline, nine (5 from the typical APD group and 4 from the atypical APD group) out of the 39 patients were taking benzodiazepines. At endpoint eight were taking benzodiazepines (3 from the typical APD group and 5 from the atypical APD group). Eight patients were on biperiden at baseline (4 from each group) and four at endpoint (2 each).

Patients' cognitive performance on the different tests ranged around 2-3 standard deviations under normative data for age and education-matched healthy Spanish population, with exception of the digit span test. [Mean whole sample of patients for TMA test = 65.20 vs. 24.40 (SD = 8.71) for normal population; Mean TMB test = 162.71 vs. 50.68 (SD = 12.36); Rey Figure = 13.44 vs. 21.48 (SD = 5.54); FAS (verbal fluency) = 25.05 vs. 38.75 (SD = 4.80); Mean for Stroop test (word/color interference) = 82.0 vs. 49 (SD = 15.5). See Ardila et al [38] for complete mean scores in Spanish population.

The baseline between-group comparison is summarized in Table 1. Differences were observed in two variables: the PANSS positive subscale score and CPZ units (both were used as covariables in repeated measures analyses). Regarding the cognitive variables at baseline, the group treated with conventional APDs showed a poorer performance in the Trail Making Test part B (*t *= 2.69; *P *= 0.01) and committed more total errors in the WCST (*t *= 2.14; *P *= 0.03). Sex distribution was equal in both groups.

**Table 1:** Baseline comparison between patients treated with conventional and atypical APDs: demographic, outcome, pharmacological, and clinical variables.

	Conventional APD		Atypical APD		*t*	*P*
	
	Mean	SD	Mean	SD		
Age	33.69	10.03	32.69	7.49	0.350	0.728

Years of education	9.15	3.33	10.73	2.76	-1.56	0.126

Age at onset	22.76	5.44	26.03	7.60	-1.37	0.176

Years of illness	10.92	8.83	6.65	5.98	1.78	0.082

Number of prior episodes	2.91	1.97	1.91	1.28	1.83	0.075

Chlorpromazine equivalent units	1116.15	708.97	602.88	264.60	3.29	**0.002**

Biperiden (mg)	1.08	1.98	0.30	0.78	1.75	0.087

Diazepam units	6.43	10.48	2.11	7.89	1.44	0.158

Number of APD	1.23	1.59	1.00	0.00	1.99	0.054

PANSS positive	17.84	4.89	10.15	2.98	6.093	**0.0001**

PANSS negative	19.69	9.12	21.11	8.82	-0.470	0.641

PANSS general	31.61	8.69	30.11	7.07	0.578	0.567

PANSS total	69.15	17.36	61.38	16.32	1.37	0.178

HRSD-21	4.87	3.56	6.12	4.97	-0.65	0.519

Premorbid adjustment	4.50	1.06	3.60	2.14	0.113	0.265

APD: Antipsychotic Drug; SD: Standard Deviation; HRSD-21: Hamilton Rating Scale for Depression, 21 items

### Clinical and neurocognitive changes in all patients

Significant differences between T1 and T2 were observed for the following cognitive measures.

A) Semantic verbal fluency, as assessed using the CIG test (*t *= -4.14; *P *< 0.000; *d *=.664) and FAS Test (*t *= -3.76; *P *= 0.001; *d *=.602).

B) Executive functions, as assessed using the WCST for total errors (*t *= 2.03, *P *= 0.04; *d *=.324) and for perseverative errors (*t *= 2.15; *P *= 0.03; *d *= 344), the Color/Word interference part of the Stroop Test (*t *= 2.77; *P *= 0.009; *d *= 0.444), and the Trail Making Test part B (*t *= 2.74; *P *= 0.009; *d *= 0.440).

C) Auditory verbal memory, as assessed using the Digit Span Forward Test (*t *= 3.89; *P *< 0.000; *d *= .620).

D) Visual memory, as assessed using the Rey-Osterrieth Complex Figure Test for immediate (*t *= -4.26; *P *< 0.000; *d *=.681) and delayed (*t *= -3.35; *P *= 0.002; *d *=.536) visual recall.

Considering the whole sample, no differences were observed between T1 and T2 for the various PANSS subscales.

The repeated measures analyses did not reveal any main effect of the patient group (conventional or atypical APDs) on any of the cognitive tests used (Table 2); however, major effects of the patient group were observed for the PANSS positive and general PANSS subscale scores (Table 3).

**Table 2:** Effect of the type of antipsychotic treatment on the evolution of cognitive performance (repeated measures analysis)

	Conventional APD				Atypical APD				F	*P*
	
	T1		T2		T1		T2			
	
	Mean	SD	Mean	SD	Mean	SD	Mean	SD		
Digit Span forward	5.58	1.11	5.16	0.98	5.92	1.29	5.30	1.22	0.014	0.906

Digit Span Backwards	3.42	0.95	3.58	0.75	3.92	1.67	3.76	1.24	0.601	0.443

WCST categories	3.15	1.72	3.75	1.92	4.15	2.46	4.46	2.12	0.155	0.697

WCST total errors	62.61	18.82	49.83	22.90	41.23	33.28	35.53	30.20	0.024	0.879

WCST perseverative errors	35.00	14.14	25.75	14.39	22.65	23.20	17.07	16.11	0.095	0.760

FAS Test	39.07	9.84	44.69	9.04	40.11	12.66	48.80	14.68	2.586	0.117

CIG Test	22.07	6.60	29.69	10.75	26.53	16.54	31.84	17.68	0.296	0.590

Trail Making part A	48.50	23.24	47.36	24.51	65.53	56.77	63.10	55.47	0.176	0.677

Trail Making part B	207.00	88.39	148.46	64.67	140.57	63.77	127.46	67.00	0.912	0.346

Digit Symbol	40.30	15.16	43.00	18.18	46.42	16.14	47.92	15.88	0.000	0.988

Stroop Color-Word trial (Interference)	86.53	28.13	66.43	17.35	79.73	42.13	71.11	35.21	0.054	0.818

Babcock Story Recall Test, Immediate memory	5.43	2.25	7.43	2.62	7.90	3.42	8.30	3.83	0.602	0.444

Babcock Story Recall Test, Delayed Recall	8.71	3.45	9.78	3.32	9.63	4.81	10.05	5.17	0.037	0.849

Rey Figure, Immediate	11.38	7.10	16.80	3.76	14.48	7.76	17.21	7.71	2.354	0.120

Rey Figure, 20 min	12.46	6.14	16.36	4.08	14.42	7.97	16.92	7.87	2.533	0.120

Finger-Tapping Test, Unimanual Left Motor Performance	53.69	10.57	57.38	16.28	58.15	21.09	66.80	12.66	1.489	0.231

Finger-Tapping Test, Unimanual Right Motor Performance	61.15	15.15	62.46	24.03	73.61	20.98	77.63	23.38	0.730	0.399

Finger-Tapping Test, Bimanual Left Motor Performance	52.92	12.02	54.07	16.13	60.80	19.93	66.75	19.21	0.113	0.739

Finger-Tapping Test, Bimanual Right Motor Performance	58.92	12.12	54.07	16.13	65.15	21.00	65.52	19.02	1.174	0.286

APD: Antipsychotic drug; DS: Standard Deviation; WCST: Wisconsin Card Sorting Test; CIG Test: Category Instant Generation Test.

**Table 3:** Effect of the type of antipsychotic treatment on the evolution of clinical measures (repeated measures analysis)

	Conventional APD				Atypical APD				F	*P*
	
	T1		T2		T1		T2			
	
	Mean	SD	Mean	SD	Mean	SD	Mean	SD		
PANSS positive	17.84	4.89	13.46	4.46	10.15	2.98	11.73	4.63	7.719	**0.009**

PANSS negative	19.69	9.12	19.23	7.60	21.11	8.82	20.19	8.62	1.067	0.309

PANSS general	31.61	8.69	32.30	8.69	30.11	7.07	28.73	8.70	9.672	**0.004**

PANSS total	69.15	1.11	65.00	18.40	61.38	17.76	60.65	18.73	3.234	0.081

HRSD-21	4.87	3.56	5.62	1.92	6.12	4.97	6.80	5.30	0.835	0.369

APD: Antipsychotic drug; SD: Standard Deviation; PANSS: Positive and Negative Symptoms Scale; HRSD-21: Hamilton Rating Scale for Depression, 21 items

### Correlational analyses

Significant correlations between outcome, clinical, and treatment variables at T1 and T2-T1 and neuropsychological change scores for the two patient groups are shown in supplemental material. Age, age at onset, length of illness, and number of prior episodes and hospitalizations did not correlate with any of the neurocognitive variables.

### Regression analyses (see table 4)

**Table 4:** Regression analysis

Dependent variable	Variables included in the regression model	**R**^2^	F	*P*
CIG (T2-T1)	*PANSS general*	0.144	7.417	0.010

TMA Test (T2-T1)	*Biperiden*	0.089	4.711	0.036

Stroop interference (T2-T1)	*PANSS negative*	0.089	4.241	0.048

WCST total errors (T2-T1)	*Diazepam*	0.092	4.871	0.034
	*Biperiden*	0.183	5.253	0.010

Digit Symbol (T2-T1)	*Biperiden*	0.130	6.666	0.014

Digit Span Backwards (T2-T1)	*EMC*	0.092	4.830	0.034

Finger-Tapping Test, Unimanual Right Motor Performance (T2-T1)	*Biperiden*	0.157	8.057	0.007

CIG Test: Category Instant Generation Test; TMA Test: Trail Making Test, part A; WCST: Wisconsin Card Sorting Test; EMC: equivalents milligrams chlorpromazine.

The type of antipsychotic medication did not predict the performance in any neuropsychological test. Dosage of biperiden significantly predicted the performance in the Stroop Test (R^2 ^= 0.132; *P *= 0.02); WCST errors (R^2 ^= 0.226; *P *= 0.01); and Finger Tapping Test, right motor performance (R^2 ^= 0.179; *P *= 0.007). Diazepam equivalent units significantly predicted the number of total errors in the WCST test (R^2 ^= 0.116; *P *= 0.03).

Concerning clinical variables, the PANSS General Psychopathology subscale significantly predicted the performance in the Category Instant Generation Test (R^2 ^= 0.167; *P *= 0.01).

## Discussion

The results from this pilot, naturalistic study suggest significant, but heterogeneous cognitive improvements for the total sample of schizophrenic subjects after two years of continuous antipsychotic treatment. Improvement was observed in the domains of semantic verbal fluency, executive functions, visual memory, and auditory immediate memory. Size effects of the differences were moderate with a more clinical relevance in the two verbal fluency tasks and visual/auditory memory (Rey figure immediate recall and Digit Span Test forward), where size effects were all over 0.6. In the opposite, after two years of treatment no cognitive improvements were observed in visual/motor processing, motor speed and working memory tasks.

As patients did not participate in psychotherapeutic interventions or programs aimed at improving cognitive function and considering the absence of significant differences between T1 and T2 in the PANSS scores, we suggest that these cognitive gains may be in part the result of continuous exposure to antipsychotic medication. Moreover, the characteristics of the sample in the present study correspond to clinically stable, chronic patients, previously stabilized by the ongoing antipsychotic medication, so the effects psychopathological changes on cognition, although not measured, are probably limited.

The relatively long time interval between the baseline and the endpoint would rule out, at least in part, learning effects associated with repeated testing [39,20].

The cognitive improvement observed in our cohort supports the results obtained in the neurocognitive component of the CATIE trial [15] and in a recent naturalistic study [40], which reported an improvement in cognition after six months of continuous treatment with APDs. The positive change described in the present study seems to exclude a potential deleterious effect of conventional APDs on a wide range of neuropsychological parameters even at the moderate-to-high dosages used here (CPZ units = 1116). As the procognitive advantages of atypical APDs reported in comparative studies, which could be caused by the use of high dosages of the drugs administered in the conventional APD group [8], remain controversial, the absence of differences in the present study can be considered more robust. Nevertheless, high dosages of conventional antipsychotics may probably impair cognition as dose reduction in patients with high dosages may lead to cognitive improvement, as shown recently [41].

Our results do not support the hypothesis of a better cognitive outcome in patients treated with atypical APD. The variable "type of APD" did not predict the improvement of performance in any of the neuropsychological tests, as assessed using regression analyses; however, intake of anticholinergic drugs seems to predict cognitive changes significantly. These results support other studies that concluded that concurrent use of anticholinergic drugs, especially their acute administration [42], is another possible explanation for the negative effects of these drugs on cognition reported in the literature [39,43]. In addition, this provides a further reason to avoid these drugs and the antipsychotics that require this sort of adjunctive medication to prevent extrapyramidal symptoms.

In contrast to the similar effect on cognitive evolution observed for both types of APDs, a significant improvement in the PANSS positive subscale score was detected in the group treated with conventional APDs. Interestingly, another significant effect, which translated into an improvement in the PANSS General Subscale score, was observed in the group treated with atypical APDs, which suggests a positive effect of these new drugs on the nonpsychotic symptoms of schizophrenia.

The limitations of the present study should be taken into account. Firstly, the relatively small sample size and the consequent lack of statistical power may have masked possible differences between the groups. In addition, these factors did not allow us to perform head-to-head comparisons of specific APDs. Secondly, as this was not a blind trial and patients were not randomly assigned to medication groups, the differences detected among groups may simply reflect a prescription bias. According to a recent meta-analysis [39], the differences in cognitive outcomes observed between conventional and atypical APDs in naturalistic, open-label studies are usually not found in double-blind randomized trials. Nevertheless, naturalistic studies have the advantage of using doses of APDs that are closer to those applied in daily practice, when compared with randomized clinical trials. The segregation of different conventional and atypical APDs into two groups represents the third limitation of this study. Although this procedure may make sense based on the clearly different mechanisms of conventional versus atypical APDs, it is also true that some intragroup differences may exist, especially among subjects taking different atypical APDs [44,12]. Finally, the cognitive improvements observed for the schizophrenic patients after two years of treatment could be questioned, as the present study lacks a control group of healthy subjects. These limitations are in part compensated by the two-year lapse between the evaluations, which is substantially longer than that of most published longitudinal studies comparing conventional and atypical APDs. This long period of continuous treatment may help to minimize learning effects, which renders eventual differences between the groups more robust. Several animal/preclinical studies have demonstrated that both conventional and atypical APDs increase neurogenesis and proliferation of nonneuronal cells in the adult brain, particularly in some areas of the hippocampus (see 45 for a review). This may result in cognitive improvement in terms of memory and learning. The translational effect of neurogenesis on cognitive gains/enhancements can only be correctly evaluated by longitudinal studies that incorporate more than a few weeks between assessments.

## Conclusions

Data from the literature showing that cognitive measures can predict functional outcomes [6] emphasize the importance of cognitive enhancement. Antipsychotic continuous treatment represents one of the few ways to remediate these deficits, albeit modestly. Therefore, strategies aimed at improving treatment adherence may help prevent cognitive decline and allostatic load [46].

Bearing in mind that this pilot study lacks a representative sample of patients, these preliminary finding are consistent with the recent literature in which atypical antipsychotics have not demonstrated cognitive benefits over typical or conventional antipsychotics. Our results did not show a clear advantage of atypical over conventional APDs on cognitive performance, however, the general improved tolerability profile of second generation antipsychotics regarding neurological side effects may facilitate treatment adherence, which in turn may result in cognitive improvement. Anyway, in the absence of other reasons to change ongoing treatment, which include negative or affective symptoms or lack of compliance with the regimen, the switch from typical APD at low or moderate doses to atypical APDs is not justified if based solely on the expectation of a more favorable cognitive outcome. Nevertheless, other psychopharmacological and psychosocial strategies should be implemented to enhance cognitive outcome in schizophrenic patients.

## Competing interests

Role of funding source

Funding for this study was provided by CIBERSAM, which had no further role in study design, the collection, analysis, and interpretation of data, the writing of the report, or in the decision to submit the paper for publication.

Dr. Vieta has received grants from or acted as a consultant for the following companies: AstraZeneca, Bristol Myers-Squibb, Forest Research Institute, Glaxo SmithKline, Janssen, Jazz Pharmaceuticals, Eli-Lilly, Lundbeck, MSD, Novartis, Organon, Otsuka, Pfizer, Sanofi-Aventis, Servier, Schering-Plough, Solvay, Takeda, United Biosource Corporation, and Wyeth.

Dr. Tabarés-Seisdedos has received grants from or acted as a consultant for the following companies: AstraZeneca, Janssen, Eli-Lilly, Lundbeck, Novartis, Pfizer, Sanofi-Aventis, and Wyeth that were deposited into research accounts at the University of Valencia.

Dr. Balanzá-Martínez has received grants from or acted as a consultant for the following companies: AstraZeneca, Boehringer Ingelheim, Bristol-Myers-Squibb/Otsuka, Janssen-Cilag, Pfizer, and Wyeth.

## Authors' contributions

All listed authors have contributed significantly to the manuscript and consent to their names on the manuscript. GSV, RTS, VBM and JSF conceived of the study, participated in its design and coordination and helped to draft the manuscript. PC collected data and carried out the neuropsychological assessments. JSM, AMA and EV revised the article critically for important intellectual content. All authors read and approved the final manuscript

## Pre-publication history

The pre-publication history for this paper can be accessed here:

http://www.biomedcentral.com/1471-244X/10/47/prepub
